# Integrative Precision Medicine for Dementia and Alzheimer's Diseases in Africa

**DOI:** 10.1016/j.nbas.2023.100095

**Published:** 2023-08-30

**Authors:** Abdullahi Tunde Aborode, Nike Jesutofunmi Idowu, Samuel Tundealao, Joseph Jaiyeola, Ezemba Constance Chinyere, Seto Charles Ogunleye, Mercy Olorunshola, Ogunware Adedayo Emmanuel

**Affiliations:** a Healthy Africans Platform, Research and Development, Ibadan, Nigeria; b Department of Chemistry, University of Nebraska Lincoln, USA; c School of Public Health, University of Texas Health Science Center at Houston, Houston, TX, USA; d Department of Demography, College for Health, Community and Policy, University of Texas at San Antonio; e Department of Microbiology, Chukwuemeka Odumegwu Ojukwu University, Uli, Anambra State, Nigeria; f Department of Neuroscience, Developmental, and Regenerative Biology, University of Texas at San Antonio, USA; g Comparative Biomedical Sciences, College of Veterinary Medicine, Mississippi State University, Mississippi State, 39760, MS, USA; h Department of Biological Sciences, State University of New York at Binghamton, NewYork, USA

Advanced medical facilities and research in North America, Europe, and certain regions of Asia have facilitated the early detection of Alzheimer's and Dementia through the utilization of brain imaging techniques and biomarkers [1]. The standard treatment approach frequently entails using a combination of pharmacological interventions and cognitive-based therapeutic techniques. Home care, assisted living, and nursing facilities are commonly utilized alternatives for providing care [1].

In the African context, the provision of dementia and Alzheimer's care is influenced by a range of problems, including but not limited to, restricted healthcare accessibility, prevailing social stigma, and deeply ingrained cultural beliefs [2]. The promotion of community-based therapies, awareness campaigns, and caregiver support groups is underway. Nevertheless, numerous African nations face constraints in terms of the accessibility of advanced medical resources and specialized care facilities [2].

Integrative precision medicine for dementia and Alzheimer's in Africa encompasses a comprehensive and interdisciplinary strategy that links diverse facets of healthcare and precision medicine to effectively tackle the distinctive obstacles and the need encountered by individuals affected by dementia in the African context. Precision medicine delivers precise treatment and healthcare services that take into account an individual's unique genetic, environmental, and lifestyle characteristics [3]. The utilization of genetic testing and biomarker analysis has proven to be valuable in the identification of individuals at risk for dementia or the diagnosis of specific types of dementia [4]. This information has the potential to inform treatment decisions and facilitate the development of tailored interventions.

Africa exhibits a wide range of cultural and socioeconomic contexts, reflecting its inherent diversity as a continent. In the context of dementia and Alzheimer's in countries like Nigeria, and South Africa, the implementation of integrative precision medicine takes into account cultural factors, beliefs, and practices in order to ascertain the suitability and efficacy of interventions within the specific local population [3].

Access to healthcare services in various African nations like Zambia, Zimbabwe, Somalia are often constrained, particularly in rural regions [4]. The primary objective of integrative precision medicine is to enhance the availability of dementia care, encompassing diagnostic services, specialized clinics, and support networks.

The provision of care for individuals with dementia and Alzheimer's in Africa necessitates the utilization of a multidisciplinary framework, which entails the involvement of healthcare practitioners from diverse fields, including neurology, psychiatry, nursing, and social work [5]. The promotion of collaboration among various professionals is imperative in the context of integrative precision medicine, as it facilitates the provision of comprehensive care and support to individuals with dementia and their families.

The consideration of lifestyle and environmental factors in different Africa countries like Ghana, Nigeria, Rwanda are imperative in the context of integrative precision medicine, as these factors have the potential to influence both the risk and progression of dementia and Alzheimer's disease [5]. This may encompass the promotion of nutritious dietary patterns, engagement in regular physical activity, provision of cognitive stimulation, and establishment of environments conducive to supporting individuals with dementia and Alzheimer's disease as supported by high income countries like UK, Germany, and USA.

In light of the geographical obstacles and inadequate healthcare infrastructure in certain regions of Africa, the utilization of telemedicine and digital health solutions holds significant potential in facilitating the provision of comprehensive precision medicine in countries like Rwanda, Nigeria, and South Africa [6]. The utilization of remote consultations, telemonitoring, and mobile health applications has the potential to enhance accessibility to healthcare services and provide valuable assistance to individuals affected by dementia and Alzheimer's disease, as displayed in countries like UK, and US [6]. The pursuit of integrative precision medicine for dementia and Alzheimer's in Africa necessitates continuous research aimed at comprehending the distinctive genetic, environmental, and socio-cultural determinants that impact the manifestation of dementia and Alzheimer's disease [7].

Investing in local research initiatives, developing research capacity, and fostering collaborations with international institutions are crucial strategies for promoting advancements in dementia and Alzheimer's care. It is imperative to enhance research capacity in Africa; however, it is crucial to adopt a balanced approach that avoids marginalizing health service providers as well as patients in maintaining an exclusive hierarchy. The principle of pragmatism is of paramount importance, and the pursuit of international collaboration, as advocated for, has the potential to effectively serve the sustainability of integrative precision medicine in dementia.

Within numerous African communities, there exists a dearth of knowledge and comprehension pertaining to dementia and Alzheimer's disease on precision medicine. The absence of sufficient awareness results in a delay in diagnosing the condition, the perpetuation of social stigma, and suboptimal utilization of the resources that are currently accessible [8]. Numerous African nations face the challenge of inadequate healthcare infrastructure, characterized by a scarcity of healthcare professionals, particularly specialists in the fields of neurology and genetics [9]. The limited availability of resources poses a barrier to the provision of precision medicine services and restricts the accessibility of cutting-edge diagnostic tools and therapies.

The lack of demographic and prevalence data represents a big hurdle to further research and interventions. The current body of research and data pertaining to dementia and Alzheimer's disease in Africa is limited, particularly in regards to genetic variations and risk factors specific to the African population. The absence of sufficient data poses a significant obstacle to the advancement of personalized treatment approaches and precision medicine interventions specifically designed for the African population [10].

Also, the role of age, sex, socioeconomic/educational status, and other chronic pathologies (diabetes, stroke, head trauma, etc.) are potential variables in dementia. Socioeconomic factors, including but not limited to poverty, restricted healthcare access, and cultural beliefs, exert a substantial influence on the adoption and execution of integrative precision medicine [11]. A significant portion of the African population lacks the necessary financial resources to access costly diagnostic procedures and therapeutic interventions that are integral to precision medicine.

Precision medicine holds the potential to yield substantial enhancements to the cost-effectiveness of healthcare [12]. Precision medicine has the potential to enhance the accuracy of diagnoses, optimize therapeutic interventions, and minimize the occurrence of adverse effects by customizing medicines based on individual genetic and molecular characteristics. This phenomenon has the potential to yield improved patient outcomes and potentially reduce overall healthcare expenditures by circumventing inefficient therapies and superfluous procedures [12]. Nonetheless, the execution of precision medicine presents several obstacles, including concerns around data privacy, regulatory barriers, and the necessity for sophisticated technology, all of which may have implications for its cost-effectiveness.

The diagnostic approaches employed differ among countries and regions, albeit typically encompassing a comprehensive amalgamation of medical history assessment, physical examinations, laboratory analyses, and imaging modalities [13]. In the African context, strategies may prioritize clinical assessment, basic laboratory testing, and point-of-care diagnostics as a result of limited resources. Telemedicine and mobile health initiatives are also significant contributors in this context [14]. Many nations frequently utilize sophisticated technology such as artificial intelligence (AI)-assisted diagnostics and genetic testing in conjunction with conventional approaches [15]. When evaluating solutions, it is crucial to take into account the healthcare infrastructure and resources that are accessible at the local level.

Africa exhibits a substantial degree of genetic diversity, characterized by a multitude of ethnic groups and genetic variations [15]. The field of precision medicine is predicated upon a comprehension of genetic profiles and their consequential ramifications in the realms of disease diagnosis, treatment, and prevention. The absence of comprehensive genetic databases that encompass the diverse African population poses significant challenges in the customization of precision medicine strategies [16]. The implementation of precision medicine frequently necessitates the acquisition, retention, and examination of genetic and personal data pertaining to individuals [16]. Nevertheless, it is imperative to acknowledge and tackle the ethical implications pertaining to data privacy, informed consent, and fair allocation of advantages within the African context. It is imperative to establish explicit guidelines and policies in order to safeguard the rights of individuals and uphold ethical standards in the realm of research.

One of the challenges faced by integrative precision medicine is the constraint of limited resources and funding. The successful implementation of this approach necessitates substantial allocation of resources, such as state-of-the-art laboratory facilities, advanced genomic sequencing technologies, and robust data analysis capabilities [17]. The implementation of precision medicine programs in Africa is hindered by significant challenges, namely limited financial resources and insufficient funding for research and healthcare infrastructure development.

Precision medicine has great promise in improving the accuracy of diagnoses by customizing medical interventions based on an individual's distinct genetic, environmental, and lifestyle variables [18]. This methodology has the potential to yield more precise diagnoses, adequate therapies, and enhanced patient outcomes. In order to optimize the advantages and enhance the well-being of patients and their families, several measures can be undertaken. These measures encompass the advancement of genetic testing capabilities, the promotion of data sharing among healthcare providers, the assurance of privacy and ethical considerations, and the provision of patient education regarding the possibilities of precision medicine. The establishment of effective partnerships among researchers, doctors, and patients will play a pivotal role in fully harnessing the capabilities of precision medicine for both diagnostic and therapeutic purposes.

Integrative precision medicine has promise in improving healthcare outcomes through the customization of treatments based on individual genetic, molecular, and environmental determinants on dementia. This has the potential to result in enhanced therapeutic interventions and decreased occurrence of negative side effects on a broader scale among the population [18]. Nevertheless, the implementation of cutting-edge technologies and tailored methodologies can result in elevated operational expenses. The challenges encompass ethical concerns pertaining to the safeguarding of data privacy, cultural variances in the acceptance of certain methodologies, and economic inequalities that may impede accessibility.

The field of precision medicine has led to significant advancements on dementia treatment. However, the successful integration and use of precision medicine depend on certain crucial factors. One important aspect to consider is the presence of local technical expertise [19]. To effectively implement precision medicine, a comprehensive comprehension of genetics, molecular biology, data analytics, and bioinformatics is needed. It is imperative for healthcare practitioners at the local level to acquire the necessary knowledge and expertise in order to effectively analyze genetic data and subsequently make well-informed judgments regarding treatment options.

Precision medicine is significantly dependent on state-of-the-art technologies such as next-generation sequencing and powerful imaging equipment [19]. The availability and accessibility of these technologies can exhibit significant variation, hence influencing the practicality of implementing precision medicine methodologies.

One of the significant obstacles that dementia treatment encounter is cultural challenges [20]. The willingness of patients to participate in precision medicine projects or disclose genetic information can be influenced by cultural beliefs and customs. It is imperative for healthcare providers to acknowledge and effectively address cultural considerations, while also ensuring that patients possess a comprehensive understanding of the advantages and potential consequences associated with their participation.

The utilization of genetic information gives rise to ethical considerations pertaining to the safeguarding of privacy, obtaining informed consent, and ensuring the security of data [21]. It is imperative to establish local standards and laws in order to safeguard patient confidentiality and promote responsible utilization of genetic data. The field of precision medicine creates substantial quantities of data that necessitate secure storage, processing, and analysis. The establishment of a resilient data infrastructure is of paramount importance in order to fully leverage the possibilities offered by precision medicine.

The factors of cost and accessibility are significant considerations in this context. The cost of advanced genetic testing and individualized treatments can be too high [22]. Ensuring equitable access to these technologies for all patients, irrespective of their socioeconomic situation, is a crucial factor to be taken into account. The topic of education and awareness is of significant importance. It is imperative to provide education to both healthcare professionals and the general public regarding the fundamental principles, advantages, and constraints associated with precision medicine. Increasing awareness has the potential to facilitate well-informed decision-making and promote active engagement.

Regulatory frameworks refer to the set of rules, regulations, and guidelines that govern a particular industry or sector. These frameworks are established by regulatory bodies or government agencies to ensure the establishment and execution of regulatory frameworks are imperative in guaranteeing the safety, effectiveness, and quality of precision medicine therapies [23]. It is imperative to modify these frameworks to suit the local context's specific conditions, while ensuring their compliance with international norms. Precision medicine frequently entails continuous monitoring and modifications to treatment strategies in response to the dynamic nature of genetic data [24]. The use of strategies to ensure sustained patient involvement and continued monitoring is crucial for achieving favorable outcomes.

To tackle these challenges, a comprehensive strategy is necessary, encompassing various aspects such as increasing public knowledge, allocating resources to enhance healthcare infrastructure and research capabilities, fostering international partnerships, and formulating policies that ensure fair and equal availability of precision medicine interventions. The involvement of a wide range of stakeholders, such as governments, healthcare providers, researchers, communities, and funding agencies, is of utmost importance in order to address these challenges and make progress in the field of integrative precision medicine for dementia and Alzheimer's disease in Africa [25]. In Africa, the overarching objective of integrative precision medicine for dementia is to integrate personalized methodologies with cultural sensitivity, enhanced healthcare accessibility, and collaborative endeavors in order to effectively tackle the escalating issue of dementia and Alzheimer's disease in the region. By customizing interventions to meet the specific needs of individuals and taking into account the unique circumstances of local communities, this approach holds promise for improving dementia care and support in the African context.

## Declaration of Competing Interest

The authors declare that they have no known competing financial interests or personal relationships that could have appeared to influence the work reported in this paper.
